# Rethinking livestock farming for artificial intelligence integration

**DOI:** 10.1093/af/vfag006

**Published:** 2026-02-11

**Authors:** Andrea Rosati

**Affiliations:** EAAP—The European Federation of Animal Science, Rome, Italy

**Keywords:** artificial intelligence, cybersecurity, data interoperability, precision livestock farming, workforce transformation

Implications

- *Holistic infrastructure redesign—*AI adoption in livestock farming requires not just adding sensors and devices to existing facilities, but rethinking barn layouts, workflows, and movement patterns to create “AI-ready” environments that optimize data capture and animal welfare.
- *Transformation of workforce roles and skills—*The shift to AI-driven management demands new professional profiles (e.g., digital field operators, AI system managers, data analysts) and continuous training to ensure both technological competence and critical interpretation of AI-generated insights.
- *Data integration and interoperability—*Effective use of AI relies on standardized, compatible data systems across feeding, health, reproduction, and monitoring processes, enabling seamless communication, real-time analytics, and improved decision-making.
- *Cybersecurity as a core operational priority—*Increased digitalization brings significant vulnerability to cyber threats, making cybersecurity policies, access control, IoT device protection, and staff awareness essential to safeguarding data, continuity, and trust.
- *Shift from reactive to predictive management—*AI allows real-time detection of health, behaviour, and environmental changes, enabling proactive interventions and replacing fixed schedules with dynamic, data-triggered workflows.
- *Expanded collaboration networks—*Digital platforms facilitate continuous, secure, and data-driven collaboration with veterinarians, nutritionists, researchers, and technology providers, fostering innovation, benchmarking, and co-development of AI solutions at local and global levels.

## Introduction

Animal husbandry is currently undergoing an unprecedented structural transformation, driven by the growing adoption of advanced digital technologies and, in particular, artificial intelligence (AI). The urgency of tackling global challenges—such as environmental sustainability, animal welfare, economic competitiveness, and food security—requires a profound revision of traditional management models, making it necessary to take a major leap in the collection, analysis, and use of data.

In this new scenario, artificial intelligence is not simply an additional tool but a true catalyst for change (Fuentes et al., 2022; Ohashi et al., 2024). Its integration requires a comprehensive redefinition of the business infrastructure, workflows, staff skills, relationships with consultants and suppliers, as well as cybersecurity measures. The livestock farming of the future is not built solely with sensors and algorithms, but with a systemic vision that can harness the potential of digitalization and make it operational in the real-life context of farming. For these technologies to have a real impact, a systemic approach—including interoperability, connectivity, and data protection—is essential (Banhazi et al., 2012; Caja et al., 2016). In this context, changes to buildings, housing, and animal routes, as well as the optimization of work organization, are not accessories but pillars of efficiency and welfare.

This article critically and practically examines the main areas of transformation that the adoption of AI requires in livestock farms. From the need for suitable technological infrastructure to continuous staff training, from the evolution of collaboration methods with external experts to the proactive management of data security, it outlines the prerequisites for a new, more resilient, predictive, and integrated animal husbandry.

Understanding and managing this change is essential to prevent innovation from remaining confined to an experimental or elitist level. Only a strategic and inclusive approach can ensure that artificial intelligence becomes a concrete resource for improving sustainability, productivity, and quality in animal production, in line with the long-term objectives of worldwide agriculture.

## Rethinking the Livestock Environment for Artificial Intelligence

The introduction of artificial intelligence in livestock farming represents a turning point, but the way it is often adopted is limited by a fundamental error: trying to adapt intelligent tools to structures and management logics born in pre-digital eras. Instead, it is necessary to design an environment conceived from the outset to host and enhance AI, thus, creating a truly intelligent, responsive, and sustainable system (Neethirajan, 2020).

The current “retrofit” approach—adapting AI to existing facilities and structures—has evident limitations. For example, environmental sensors and behavioral monitoring systems installed in traditional barns often suffer from coverage issues, interference, or incomplete data collection. The result is suboptimal interpretation of animals’ behavioral and physiological signals (Berckmans, 2017b).

By contrast, designing buildings and processes for AI from the planning stage means integrating the technology from the outset. This is the case in some pilot farms in the Netherlands, where barns have been constructed with layouts optimized for continuous data capture (Fielke et al., 2020). Examples include mandatory passages for robotic milking, floors with integrated pressure sensors, and adaptive lighting based on circadian rhythms—concrete examples of “AI-ready” infrastructure.

AI is not a mere plug-in. For it to deliver its transformative potential, it is necessary to create an appropriate context in terms of infrastructure, workflows, training, and organizational design. Only in this way can artificial intelligence become an ally for animal welfare, production efficiency, and environmental sustainability. Continuing to operate in structures conceived in an analogue paradigm means greatly limiting the scope of innovation.

## Structural Modifications for AI

The design of internal spaces should follow animals’ natural movement patterns, reducing stress and promoting fluid movement—especially important with mechanization, which is both enabled and required by AI. Space per animal, shading, comfortable flooring, and adequate ventilation are key factors for health and natural behavior (Telezhenko and Bergsten, 2005; Von Keyserlingk et al., 2009). A well-designed barn enables more efficient logistics: animal handling is no longer stressful or labor-intensive, but flexible and predictive—a necessary condition for integrating AI and automated systems that can promptly respond to animals’ needs (Halachmi et al., 2019).

Effective AI implementation in livestock systems also requires robust adaptation of the technological infrastructure, particularly regarding sensors and Internet of Things (IoT) devices. These tools are essential for continuous, real-time monitoring of environmental and zootechnical parameters, fundamental for data-driven management and precision farming (Table 1).

**Table 1.** AI-ready livestock ecosystem

Component	Function	Examples	Benefits
Environmental sensors	Detection of temperature, humidity, air quality	IoT thermometers, gas sensors	Prevention of heat stress, animal welfare
Wearable devices	Individual monitoring	RFID collars, accelerometers	Early detection of health issues
Computer vision systems	Behavioral analysis	HD cameras, image recognition systems	Reduced manual intervention, data accuracy
Advanced connectivity	Real-time data transmission	4G/5G, LoRa, fibre optics	Remote access, systems integration
Backup systems	Operational continuity	Generators, UPS	Prevention of critical downtime

The first essential step is installing environmental sensors capable of detecting variables such as temperature, humidity, and air quality. These data are crucial not only for assessing animal welfare but also for preventing and mitigating risks linked to environmental stress.

In parallel wearable technologies—such as electronic tags or smart collars—must be adopted for individual animal monitoring. These devices enable continuous collection of information on physical activity, rumination, reproductive status (e.g., oestrus detection), and health conditions. This level of individual monitoring allows early detection of anomalies and supports precision management strategies.

Additional technologies, such as automatic scales, RFID readers, and computer vision systems, are becoming increasingly relevant. These tools facilitate automated data collection, reduce human error, and improve the accuracy of performance monitoring and phenotyping.

The generation of large volumes of high-frequency data requires reliable data transmission networks. A stable and fast internet connection is a fundamental prerequisite, particularly critical in rural or remote areas. Consequently, investments in broadband infrastructure (fibre optics, 4G/5G networks, or low-power long-range networks like LoRa) are necessary. Poor connectivity is a significant obstacle to the large-scale adoption of AI-based services in livestock production.

Beyond connectivity, it is essential to ensure that all devices and systems are interoperable and capable of transmitting data in real time to a cloud platform or local server. This requires integrated and standardized digital infrastructure to facilitate a continuous data flow and effective analysis.

Every animal must have a stable, recognisable identity over time, and machines, barn areas, and daily operations must also be clearly coded.

Data management, however, does not end with collection and organization. For information to be truly useful, the various farm systems—dedicated to feeding, health, reproduction, and milking—must be able to communicate with each other. Too often, these systems remain isolated. The adoption of compatible, open software equipped with APIs is essential to create a cohesive digital ecosystem (Habib et al., 2024).

This approach also enables data sharing with external stakeholders—consultants, health authorities, or databases—enhancing transparency and traceability (FAO[mdash]Food and Agriculture Organization, n.d.a).

Another crucial aspect concerns data storage. Security and accessibility must go hand in hand: cloud systems or local servers with regular backups ensure continuity even in the event of failures. Furthermore, it is vital to establish different access levels, in compliance with data protection regulations such as GDPR.

However, merely collecting and storing data is not enough. It is essential to interpret and enhance them. Intelligent dashboards are an effective tool for turning numbers into operational decisions. AI models, fuelled by updated and consistent data, can progressively learn and improve their predictive capacity.

Furthermore, in rural contexts where power outages may occur, backup energy systems—such as generators—should be available to ensure uninterrupted operation of sensors and data transmission infrastructure. Similarly, redundant internet solutions should be considered to guarantee stable connectivity even in critical conditions.

In conclusion, the adoption of AI-based technologies in livestock systems depends not only on the availability of advanced analytical tools but above all on the readiness of the underlying technological infrastructure. Without adequate technological foundations, the potential benefits of AI and IoT applications in livestock farming risk remaining purely theoretical. Therefore, addressing infrastructural challenges is a strategic priority for the digital transformation of the livestock sector.

## Staff Training

Contemporary livestock farming is at the centre of a profound transformation, driven by the growing availability of digital technologies and the need to make farms more efficient, sustainable, and accountable. In this scenario, data digitalization and standardization emerge as essential elements to address sector challenges with innovative and predictive tools.

The first major step is the systematic and automated collection of data. It is now evident that relying on paper records or fragmented Excel files is no longer sustainable. Farmers and staff members must be able to rely on integrated digital systems that automate information collection, reducing human error and improving data reliability. For example, IoT sensors make it possible to monitor, in real time, key parameters such as environmental temperature, humidity, feed intake, or rumination behavior (Berckmans, 2017a).Cameras and microphones can now detect abnormal behaviors or vocalizations that may indicate stress or discomfort, while wearable devices, such as RFID collars or accelerometers, allow constant individual tracking. In this way, each animal becomes a continuous source of information, making uninterrupted monitoring of its condition possible (Caja et al., 2016).

However, the quantity of collected data is not enough. It is essential for staff to be efficient in acting properly that information is organized according to standardized formats compatible with modern analytical tools (Habib et al., 2024). Structuring data using shared criteria—such as JSON, XML, or CSV—makes it possible not only to store them in an orderly fashion but also to feed them into AI algorithms. Classifying information according to international livestock ontologies, such as those proposed by ICAR, EFSA, or FAO, ensures a common language among farmers and staff members, consultants, geneticists, and health authorities.

Technologies, no matter how advanced, do not create value on their own if they are not properly understood, interpreted and consciously used by those who operate daily within production and decision-making processes. Operators therefore need to be supported through a development pathway that goes beyond basic technical instruction or the purely instrumental use of digital tools. It is essential to build skills that enable staff to understand the meaning of the data being collected, to assess their quality and reliability, and to critically interpret the analyses generated by intelligent systems. Only in this way can data move from being simple technological outputs to becoming genuine knowledge assets that support informed decision-making (Wathes et al., 2008; Berckmans, 2017b).

A concrete and up-to-date example of this approach could be found in the *data literacy* and *AI awareness* programmes that have been introduced in recent years by many agri-food and livestock companies. Training has not been limited to learning how to use digital platforms, but has included dedicated modules on interpreting key indicators, understanding predictive models and critically discussing the outputs generated by AI algorithms, highlighting their assumptions, limitations and potential biases. In this perspective, training becomes a strategic asset: not merely a cost, but a necessary investment to integrate technology with human expertise, foster a data-driven culture and ensure that digital innovation translates into concrete, sustainable and long-lasting improvements.

The livestock farming of the future will no longer rely on disorganized notes and closed systems. It will be increasingly oriented towards the strategic use by farmers and staff members of information, capable of predicting, preventing, and planning with precision. Data digitalization and standardization are not a passing trend but the foundation of a new era in which artificial intelligence becomes a valuable ally for humans and animals alike (Fuentes et al., 2022).

## Workflow Changes

The introduction of artificial intelligence into the livestock sector is not merely a technological matter. First and foremost, it is a cultural and organizational transformation that profoundly affects workflows, requiring a rethinking of daily operations and responsibilities within the enterprise (Rohan et al., 2023). What until recently was based on direct experience and fixed schedules must now adapt to a new logic driven by data, responsiveness, and prediction (Rosati, 2024).

Traditional farming processes—often linear and structured around fixed routines, such as weekly health checks or monthly reproductive visits—are giving way to more dynamic and adaptive workflows. AI, integrated into farm systems, can provide real-time signals: abnormal behavior, an increase in heat stress, suspected oestrus, or an imminent health risk become triggers that activate targeted, immediate, proactive actions (Abbas et al., 2025; Table 2).

**Table 2.** Workflow evolution

Traditional process	New AI-driven approach	Benefits
Scheduled health checks	Continuous monitoring and alert-based intervention	Reactivity, disease prevention
Manual data recording	Automated collection via sensors	Reduced errors, real-time updates
Standard operational roles	Digital profiles (data analyst, AI manager)	Higher efficiency, strategic use of data

One of the most visible changes is the automation of repetitive operations. Monitoring, recording, environmental checks, and feed distribution no longer require constant, direct human intervention. Sensors and intelligent systems now detect, record, and interpret data. The human role is to supervise, validate, and intervene only when necessary. This is a silent but profound revolution: it lightens the operational burden but requires new skills.

Action, once the farmer’s first instinct when faced with a perceived problem, now becomes the last step in a complex chain that begins with data collection and analysis. Before administering treatment to an animal that “looks sick,” activity, intake, temperature, and vocalization indexes are examined. Only if this set of parameters confirms a risk pattern does intervention occur (Rohan et al., 2023; Abbas et al., 2025). This is a more reflective, rational form of livestock farming, focused on verification rather than impression.

This transformation generates new daily tasks. Each day, the operator must consult farm dashboards, analyse AI-generated alerts, and verify any deviations in consumption, production, or behavior. Corrective actions are no longer tied to fixed schedules or shifts but are triggered when data indicate the need. Regularity gives way to adaptability; predetermined time slots to emerging priorities.

Roles within the enterprise change significantly. New profiles emerge: the digital field operator, the AI systems manager, the technician with analytical skills. Barn work becomes less execution-oriented and more strategic, less focused on repetitive tasks and more on intelligent monitoring, critical analysis, and interpretation. Internal and external collaboration is also strengthened: workflows extend beyond the farm perimeter to consultants, veterinarians, geneticists, nutritionists, and regulatory bodies. Collected and interpreted data circulate within a network that unites different actors in a single digital supply chain.

Technical interventions are also reorganized according to new logic. Actions are no longer based on habit or fixed cycles but on priorities objectively generated by systems. A feed order is no longer placed when stock runs out but when AI detects an upward consumption trend. A treatment is no longer planned for Monday morning but when a biological or behavioral signal justifies it.

Finally, documentation processes are transformed. Health reports, traceability data, and indicators required by control systems are produced automatically by software, updated in real time, and validated by operators without the need for manual transcription. Even livestock bureaucracy becomes intelligent, lean, and transparent.

In summary, the shift to AI-driven workflows marks a paradigm change: from manual collection to continuous sensor data, from instinct-based decisions to data-based ones, from rigid management to reactive and predictive systems, from execution-focused roles to specialized profiles in supervision and interpretation. It is a profound evolution that requires vision, training, and trust in data science (Rosati, 2024)—a transformation which, if managed well, promises a more efficient, sustainable, and resilient livestock farming.

## Data Security Management (Cybersecurity)

When a livestock business decides to embrace artificial intelligence, digitizing processes and automating data collection, it also faces a new type of vulnerability: cyber threats. The adoption of technologies such as connected sensors, cloud-based management systems, robotic milking or feeding equipment offers tremendous opportunities for efficiency, but at the same time opens doors which, if not properly secured, can become entry points for unauthorized access. Cybersecurity thus becomes a strategic element for the survival and competitiveness of the business (Kshetri, 2020).

Cybersecurity in livestock farming is no longer an issue to be postponed. Although historically less digitized than other production sectors, livestock enterprises are today increasingly interconnected—and therefore, more exposed. The first line of defence is the protection of the digital infrastructure: updated enterprise firewalls, antivirus, and antimalware software installed on every connected device—from PCs to company smartphones, from servers to routers—are essential measures (Stallings, 2018). These must be combined with systematic updating: every operating system, management software, or IoT device firmware should be kept at its latest version to prevent known vulnerabilities from being exploited in attacks.

Another critical point is access management. Cybersecurity is not only about machines but also—and above all—about people. Risky practices such as shared accounts or simple, recurring passwords must be abandoned. Every operator must have an individual account with access rights calibrated to their role. Where possible, two-factor authentication (2FA) should become standard practice, especially for remote access to cloud systems or management applications (NIST, 2022).

When it comes to the cloud, protecting stored data is one of today’s biggest challenges. Using certified platforms compliant with, for example, the rules established by the European GDPR is not enough: clear contracts with software providers are needed, defining who owns the data, how it is stored, which security measures are applied, and who can access it. Good corporate data governance always starts with contractual transparency.

However, security does not end with prevention. Even the most cautious businesses must have a plan to deal with the unexpected. Performing periodic backups—on separate media and, preferably, in the cloud—is essential to ensure operational continuity. But backup alone is not enough: the ability to restore data in case of failure or attack must be tested regularly. Without disaster recovery tests, a backup is just the illusion of security (Cichonski et al., 2012).

Another often underestimated aspect is the protection of IoT devices. Every sensor, camera, automatic scale, or weather station is a potential entry point for malicious actors. For this reason, factory credentials should be changed, unused ports deactivated, and direct internet exposure limited in favor of local networks or secure Virtual Private Networks (Sicari et al., 2015).

But technology alone is not enough. A simple phishing email or an infected USB stick can undermine even the most sophisticated defences. Investing in staff training is, therefore, crucial: teaching them to recognize signs of cyber-attacks, to use company devices correctly, and to know how to respond in case of suspicion is a corporate responsibility, not an optional extra (Hadnagy, 2018).

All of this must be integrated into a coherent framework: a corporate cybersecurity policy, drafted with the support of a competent contact person—internal or external—defining rules, procedures, obligations, and emergency plans. Only in this way can the adoption of AI in livestock farming realize its full potential without exposing the company to silent but devastating risks. In a context where data is the new capital, protecting it means protecting the enterprise, its reputation, and its future. Cybersecurity is no longer a specialist matter: it is a shared responsibility that begins every day, with every click (Von Solms and Van Niekerk, 2013).

Finally, in the shift toward AI-integrated livestock farming, data governance is far more than a technical checkbox; it is the ethical and operational foundation of the entire movement. As farms transform into interconnected, data-driven hubs, having clear rules for ownership, privacy, and security is vital to protect sensitive details like animal health records and specialized breeding data. Strong governance does more than just protect information—it builds the trust necessary for stakeholders to share the high-quality, standardized data needed to train reliable AI models (Jouanjean et al., 2020). By prioritizing accountability and clear standards from day one, farmers can guard against risks like data leaks or biased algorithms, ultimately making AI a tool for a more sustainable and profitable future.

## Collaboration with Experts and External Partners

The introduction of artificial intelligence into livestock farming is not only a technological evolution but marks a radical transformation in how farms collaborate with experts and external partners. This change entails a shift from episodic and reactive management to continuous, predictive, and integrated management, in which collaboration becomes an open, interconnected digital ecosystem (Wolfert et al., 2017).

One of the main changes concerns the way technical consultants (veterinarians, nutritionists, geneticists, agronomists) interact: they will no longer operate exclusively in person, but will be actively and in real time involved via digital platforms and cloud portals This digital integration will enable experts to directly access farm data, analyse trends, configure alerts, and provide timely operational recommendations (Rutten et al., 2013).

In this new paradigm, structured and secure data sharing will be central. External partners will be able to access both historical and real-time datasets, increasing the accuracy and timeliness of analyses. A paradigmatic example is the veterinarian who can detect a health problem before it becomes clinically apparent, thanks to constant monitoring of physiological parameters (Neethirajan, 2020).

The adoption of AI will also require an evolution in the role of experts: no longer mere providers of solutions, but interpreters of results generated by algorithms, co-authors of business decisions. This will require specific training and a new level of digital literacy for all actors involved (Eastwood et al., 2019).

Alongside traditional consultants, new professional roles will emerge—data analysts, system integrators, AI developers, cybersecurity experts—who will contribute to the development, customization, and security of intelligent systems. Collaboration with suppliers will become results-oriented, measurable through farm-collected data, and no longer-based solely on product provision (Fielke et al., 2020).

Finally, livestock enterprises will increasingly be involved in collaborative networks and applied international research projects. This will enable anonymous data sharing, participation in benchmarking systems, access to advanced services, and direct influence on the development of new AI technologies (Bronson and Knezevic, 2016). These relationships, however, will require a thorough revision of contractual agreements to regulate access, intellectual property rights, confidentiality, and protection of sensitive data (Carbonell, 2016).

In conclusion, AI adoption transforms collaboration with external experts into a strategic and ongoing partnership, based on shared data, remote access, multidisciplinary expertise, and co-development of digital solutions. This is a crucial step in ensuring that livestock farming at global level achieves more efficient, sustainable, and innovation-driven management.

## Conclusions

The introduction of artificial intelligence into livestock farming cannot be considered a mere technological upgrade; it must be interpreted as a systemic transformation of the entire production model. The adoption of AI entails a profound revision of business organization, required skills, relationships with consultants and suppliers, as well as digital infrastructure and cybersecurity protocols (Eastwood et al., 2019; Klerkx et al., 2019).

To achieve intelligent and sustainable livestock farming, AI adoption must be based on a synergy between physical infrastructure designed for animal welfare (ventilation, materials, microclimate), environments structured to facilitate work organization, and optimized spaces and routes for animal mobility and health. This integration of essential elements allows technology not only to operate but to truly transform livestock farming into a resilient, productive sector.

For this transition to generate real value, it must be approached strategically, combining technological innovation with management vision. Businesses must invest not only in intelligent tools but also in staff training, data standardization, system interoperability, and the creation of collaborative ecosystems open to research and dialogue with other actors in the supply chain (Wolfert et al., 2017; Fielke et al., 2020). Knowledge management becomes a key factor, where the ability to translate data into operational decisions is closely linked to digital literacy and organizational adaptation.

Only in this way can artificial intelligence become an effective ally for improving sustainability, productivity, and animal welfare, transforming livestock farming from a traditional sector into a leading player in the agri-food innovation (Bronson and Knezevic, 2016; Neethirajan, 2020). If adopted systemically and inclusively, intelligent technologies can help reduce environmental impact, optimize resource use, and improve transparency along the production chain.

Ignoring these opportunities—or worse, underestimating the complexity of the change required—would mean relinquishing a strategic competitive advantage and condemning the sector to a marginal role in the future of agriculture. Conversely, managing this transformation means building a more efficient, transparent, responsive, and resilient livestock system, ready to face the challenges of the 21st century with scientific and sustainable tools (Carbonell, 2016; Klerkx et al., 2019).

## Author Biography

Andrea Rosati have earned degrees in Animal Sciences from the University of Perugia (Italy), alongside an MSc in Animal Genetics and a PhD in Biometrics and Animal Genetics from the University of Nebraska—Lincoln (USA). His career commenced at the Italian Association of Animal Breeders (AIA) where he served from 1993 to 2001, ultimately ascending to the role of National Technical Manager. Andrea’s influence extends across numerous roles, including his tenure as Director-General of the Italian Laboratory of Animal Genetics. Currently, he holds the distinguished position of Secretary-General for both the European Federation of Animal Science (EAAP) and the World Association for Animal Production (WAAP). He also had been leading the International Committee for Animal Recording (ICAR) for an 11-year period, during which he played a key role in shaping global standards for animal identification, performance recording, and genetic evaluations. In his capacity as a project coordinator, Andrea led two EU-funded international research projects and has contributed to over other 20 additional EU-funded international research projects. His expertise has been showcased through lectures in over 40 countries, particularly focusing on animal production and sustainability. Andrea co-founded four animal science journals and has authored numerous research articles and dissemination pieces, in addition to writing book chapters centered on animal science. Presently, he directs EAAP and WAAP, leading therefore a team that is instrumental in giving services to a very large animal science community by, for example, coordinating international scientific conferences, webinars, workshops, and research projects within the realm of animal science.
